# Nitrogen Fertilization Effects on Productivity and Nitrogen Loss in Three Grass-Based Perennial Bioenergy Cropping Systems

**DOI:** 10.1371/journal.pone.0151919

**Published:** 2016-03-18

**Authors:** Brianna E. L. Duran, David S. Duncan, Lawrence G. Oates, Christopher J. Kucharik, Randall D. Jackson

**Affiliations:** 1 Department of Agronomy, University of Wisconsin, Madison, United States of America; 2 DOE—Great Lakes Bioenergy Research Center, University of Wisconsin, Madison, United States of America; USDA-ARS, UNITED STATES

## Abstract

Nitrogen (N) fertilization can greatly improve plant productivity but needs to be carefully managed to avoid harmful environmental impacts. Nutrient management guidelines aimed at reducing harmful forms of N loss such as nitrous oxide (N_2_O) emissions and nitrate (NO_3_^-^) leaching have been tailored for many cropping systems. The developing bioenergy industry is likely to make use of novel cropping systems, such as polycultures of perennial species, for which we have limited nutrient management experience. We studied how a switchgrass (*Panicum virgatum*) monoculture, a 5-species native grass mixture and an 18-species restored prairie responded to annual fertilizer applications of 56 kg N ha^-1^ in a field-scale agronomic trial in south-central Wisconsin over a 2-year period. We observed greater fertilizer-induced N_2_O emissions and sub-rooting zone NO_3_^-^ concentrations in the switchgrass monoculture than in either polyculture. Fertilization increased aboveground net primary productivity in the polycultures, but not in the switchgrass monoculture. Switchgrass was generally more productive, while the two polycultures did not differ from each other in productivity or N loss. Our results highlight differences between polycultures and a switchgrass monoculture in responding to N fertilization.

## Introduction

Nitrogen (N) pollution from agricultural systems has local, regional, and global environmental impacts. Nitrate (NO_3_^-^) leaching can negatively impact drinking water quality [1] and contributes to eutrophication events such as the Gulf of Mexico hypoxic zone [2,3]. Ammonia volatilization contributes to acid precipitation, while nitrous oxide (N_2_O) is the single greatest ongoing source of ozone depletion [4] and an extremely potent greenhouse gas [5]. These forms of N pollution are commonly linked to excessive or misapplied N fertilizer [6,7]. To combat this, nutrient application guidelines and best management practices have been developed for many major cropping systems (e.g. [8]). These guidelines rely on knowledge about a cropping system and its performance under diverse conditions; however, such knowledge may be lacking for novel cropping systems such as perennial polycultures. Generating data on N dynamics and idiosyncrasies is thus a key element in the development of a novel cropping system.

The ongoing development of cellulosic (i.e. “second generation”) biofuels has spurred interest in the use of diverse mixtures of perennial species as biomass feedstock cropping systems [9]. Second generation biofuels use cellulose and other non-edible plant materials, in contrast to first generation fuels, which are produced from edible sugars, starches, or oils [10]. This creates the possibility of commercializing land covers, such as diverse assemblages of tallgrass prairie species, which have previously been valued purely for their aesthetic and ecological properties [11]. Despite their benefits, these systems lack the wealth of agronomic knowledge available for more conventional crops [12]. A growing body of research and experience informs management of certain biomass feedstock species, most notably switchgrass (*Panicum virgatum*) [13,14], but knowledge of the agronomic behaviors of polycultures in bioenergy feedstock cropping systems is much less developed [15]. As fertilization will likely be necessary for sustaining high levels of productivity in these systems [16], it will be critical to understand the agronomic and ecological dynamics of polyculture cropping systems.

A sizeable body of ecological research and theory explores the role of plant community diversity and composition in ecosystem functioning. Many studies find increasing the number of species present correlates to greater plant productivity [17,18], although multispecies assemblages rarely out-produce monocultures of highly productive species [19]. In some cases, increasing the number of species and functional groups in a plant community decreases soil N_2_O emissions [20,21] and reduces soil NO_3_^-^ pools [22,23]. The effect of species diversity on ecosystem dynamics is frequently attributed to complementarity, where differences in species-specific resource utilization result in more complete exploitation of ecosystem resources [24,25]. Nutrient management strategies designed for particular species in monoculture may thus yield different results in diverse species assemblages.

In this study, we explore how nitrogen fertilization impacts N loss pathways and plant productivity in three biomass production systems based on grasses and forbs native to North America. The systems we studied were a switchgrass monoculture, a mixture of five grasses, and a mixture of 18 prairie species. All systems were managed at agronomic scale and split into plots receiving either replacement-level N fertilizer or no fertilizer. For each system, we measured aboveground productivity, N_2_O emissions, and potential NO_3_^-^ leaching, with the aim of determining how these properties were impacted by N fertilization in the three systems.

## Materials and Methods

### Field sites

The study was conducted at the Great Lakes Bioenergy Research Center’s Biofuel Cropping Systems Experiment (BCSE) at the Arlington Agricultural Research Station (Arlington, WI, 43°17’45” N, 89°22’48” W, 315 masl) during the 2011 and 2012 field seasons. Predominant soils are classified as Plano silt loam (Fine-silty, mixed, superactive, mesic Typic Argiudoll). Mean annual air temperature and precipitation are 6.8°C and 869 mm respectively [26]. Cumulative precipitation and average daily temperature for 2011 and 2012 are given in the supporting information (S1 Fig).

The BCSE was established in 2008 and contains eight cellulosic biofuel feedstock production systems managed at agronomic scale in a randomized complete block design with five replicates (five blocks). We selected three blocks where other studies were conducting more intensive biophysical parameter modeling from which to sample. From the eight cropping systems, we selected three systems that formed a spectrum of species diversity: monoculture switchgrass (var. ‘Cave-in-Rock’), a mixture of five grasses native to the region (“native grasses”), and a mixture of 18 grass and forb species native to the region (“restored prairie”, see Table 1 for composition and diversity details). Switchgrass and mixed grass plots received early-season weed management through 2011; switchgrass received weed management again in 2012. The restored prairie received weed control in its establishment year, but none in subsequent years.

**Table 1:** Seeded species and measures of realized diversity and composition in three biofuel feedstock production systems.

	Switchgrass	Native grasses	Restored prairie
Species richness	3.6 ± 1.9	6.0 ± 2.0	10.9 ± 1.7
Shannon’s diversity index	0.7 ± 0.4	1.4 ± 0.3	1.9 ± 0.2
C3 grass cover (%)	5.0 ± 11.0	72.7 ± 29.8	90.2 ± 22.7
C4 grass cover (%)	127.2 ± 49.2	103.2 ± 47.5	21.1 ± 22.5
Non-legume forb cover (%)	11.9 ± 18.8	6.8 ± 11.3	86.5 ± 24.9
Legume cover (%)	none observed	none observed	10.4 ± 14.2
Seeded species	*Panicum virgatum* L.	*Panicum virgatum* L.	*Panicum virgatum* L.
		*Andropogon gerardii* Vitman	*Andropogon gerardii* Vitman
		*Elymus canadensis* L.	*Elymus canadensis* L.
		*Schizachyrium scoparium* (Michx.) Nash	*Schizachyrium scoparium* (Michx.) Nash
		*Sorghastrum nutans* L.	*Sorghastrum nutans* L.
			*Koeleria macrantha* (Ledeb.) Schult.
			*Anemone canadensis* L.
			*Asclepias tuberosa* L.
			*Baptisia alba* (L.) Vent. var. *macrophylla* (Larisey) Isley
			*Desmodium canadense* (L.) DC.
			*Lespedeza capitata* Michx.
			*Monarda fistulosa* L.
			*Oligoneuron rigidum* (L.) Small var. *rigidum*
			*Ratibida pinnata* (Vent.) Barnhart
			*Rudbeckia hirta* L.
			*Silphium perfoliatum* L.
			*Solidago erecta* Pursh
			*Symphyotrichum novae-angliae* (L.) G.L.Nesom.

Cover measurements allowed multiple species hits per point; functional group cover is summed over all species and can thus exceed 100%. Values are means ± standard deviation.

Plots were 27 m wide × 43 m long (0.12 ha), with a 17 m wide main plot and two 5 m wide subplots (S2 Fig). The switchgrass and mixed grass main plots as well as the restored prairie subplot received annual applications of 56 kg ha^-1^ N as ammonium nitrate. Fertilizer was applied with a vacuum spreader when C4 grasses were 30–45 cm tall (2011-05-27 and 2012-05-11) in keeping with University of Wisconsin recommendations for management of established switchgrass stands [8]. The restored prairie main plot and the subplots of switchgrass and native grasses received no fertilization after establishment. The BCSE was established in spring 2008 and all systems have been harvested to a residual stubble height of 10 cm annually since 2009. We sampled at three randomly positioned 1.5 × 1.5 m quadrats (subsamples) within both main and subplots. Quadrats remained in place during the growing season, but were repositioned in the subsequent year (S2 Fig).

### Nitrous oxide emissions

Soil N_2_O fluxes were measured twice monthly from 9 May to 14 September 2011 and 7 May to 6 September 2012. N_2_O measurement and flux estimation largely followed the approach of Oates et al. [27]. Briefly, 27.15 cm diameter stainless steel non-through flow chambers were inserted 5.5 cm into the soil to an effective height of 17.3 cm for a headspace volume of 10 l. Chambers were placed in the center of marked quadrats. Plants growing within the chambers were clipped flush with the chamber top at least 24 h before measurement to allow lid closure [28]. Gas was measured by sealing the chamber and sampling headspace gas at 10 minute intervals for 30 minutes. Gas concentrations were measured by gas chromatography with a 15 mCi ^63^Ni electron capture detector for N_2_O (Agilent 7890A GC system) and infra-red gas analyzer for CO_2_ (LI-COR LI-820 CO_2_ analyzer).

N_2_O fluxes were estimated through the HMR method [29], implemented in the R package 'HMR' (v0.3.1, [30]). We previously found the HMR method to be more appropriate than linear regression at long chamber deployment times [31]. Package recommendations were followed, with secondary visual inspection for samples where linear and nonlinear fluxes differed by ≥ 0.01 mg N_2_O-N m^-2^ h^-1^. Linear interpolation between measurement dates was used to estimate cumulative N_2_O emission over the growing season. Emissions factor (EF), the percentage of fertilizer N released as N_2_O, was calculated as:

$$EF=\frac{{N_{2}O}_{F}-{N_{2}O}_{U}}{F_{N}}\%$$

where N_2_O_F_ and N_2_O_U_ are cumulative N_2_O emissions (kg N ha^-1^) for adjacent fertilized and unfertilized subplots respectively and F_N_ is the nitrogen content (kg N ha^-1^) of fertilizer applied [32,33].

### Soil inorganic nitrogen

We sampled extractable soil NO_3_^-^ at the 50–80 cm depth. This was interpreted as potentially leachable NO_3_^-^, as root biomass and plant nutrient uptake are concentrated in the top 50 cm of soil [34,35]. We sampled periodically throughout the growing season in both years (S3 Fig), but focused our analysis on samples taken near peak plant biomass. The N content of C4 grasses reaches a maximum concurrent with peak biomass [36], so our samples should represent N that escaped below the rooting zone during the period of greatest plant N utilization.

Three soil samples were removed from each quadrat and composited. Samples were homogenized and sieved to 2 mm, with rocks and biomass removed. Within 48 h of field sampling, 10 g soil samples were extracted with 2 *M* KCl [37]. Extracts were frozen prior to colorimetric NO_3_^-^ determination (according to USEPA Method 353.2, O-I-Analytical Method #2648) using a Flow Solution 3100 segmented flow injection analyzer (OI Analytical, College Station, TX). Soil subsamples (15 g) were weighed, oven-dried for 48 h at 60°C, and then reweighed to obtain soil gravimetric water content.

### Ancillary environmental measurements

Soil temperature and moisture were measured concurrently with trace gas sampling to identify potential abiotic drivers of flux differences among systems [38,39]. Soil temperature measurements were taken with a 10 cm probe (Checktemp 1C, Hanna Instruments, Smithfield, RI); soil volumetric water content was measured using a 6 cm ML3 ThetaProbe sensor (Dynamax, Houston, TX). In both cases, we took 3 measurements within 25 cm of the sampling chamber and averaged them. Volumetric water content was converted to water-filled pore space (WFPS) using bulk densities measured at the plot level in 2008 and a standard soil particle density of 2.65 g cm^-3^. WFPS is commonly linked to microbial nitrification and denitrification [40].

### Aboveground productivity and biomass properties

After all field measurements were completed (23 to 30 September 2011 and 21 to 27 August 2012), all aboveground biomass from each quadrat was hand-harvested to ground level and then dried and weighed to estimate aboveground net primary productivity (ANPP). ANPP peaked earlier in 2012 due to above-average March temperatures and an early summer drought (S1 Fig). Plant species cover was measured immediately prior to harvest using the point-intercept method [41]. Each quadrat was divided into 49 evenly-spaced points at which each unique plant species intercepted by a dropped rod was recorded. The percent cover for each species was estimated as the percentage of points at which the species was observed. Multiple species could be counted at each point, so percent cover summed over all species could exceed 100% [41]. In addition to quadrat-level data, entire plots (main and subplot) were harvested to 10 cm stubble height following a killing frost (10 October 2011 and 7 November 2012). Total N content of a representative subsample of the harvested material was determined by combustion using a Flash EA 1112 Automated Elemental Analyzer (Thermo Finnigan, Milan, Italy).

### Statistical analysis

Data analysis was conducted in the R statistical environment (v3.1.1, [42]). Values for N_2_O emissions and NO_3_^-^ concentrations were log-transformed prior to analysis to meet assumptions of normality. Negative NO_3_^-^ concentrations were replaced with half the smallest positive concentration observed (0.04 μg N g^-1^ dry weight soil). All analyses used linear mixed effect models from the R package 'nlme' (v3.1.1, [43]). We employed a nested random effects structure of subsample within subplot within plot within block (S2 Fig) to properly account for lack of independence in our data. We allowed variances to differ among treatments, identifying the most parsimonious grouping structure by likelihood ratio comparison of nested models. Significance of treatment differences was done separately by year using the R package 'lsmeans' (v1.06, [44]) and a Tukey-corrected *P* < 0.05 cutoff.

## Results

### Fertilization increased nitrous oxide emissions more in switchgrass than in polycultures

**Fig 1:**
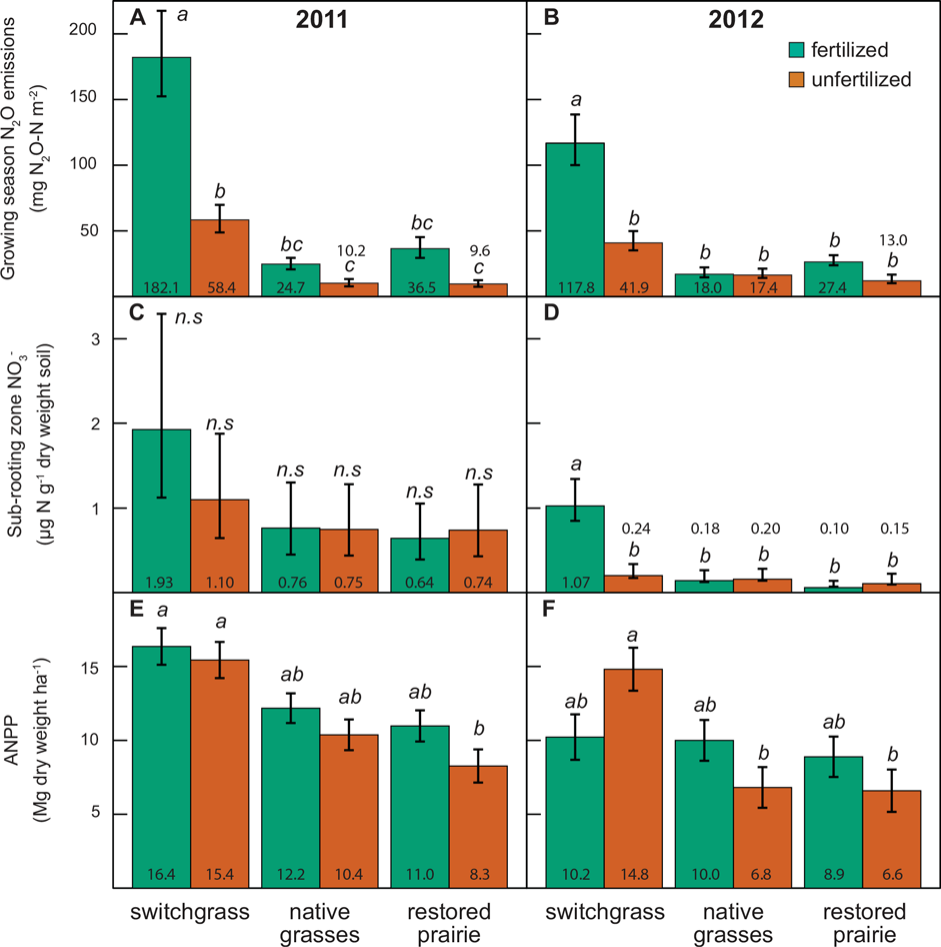
Average N losses and aboveground net primary productivity (ANPP) for each crop and fertilizer treatment in 2011 and 2012. Within each panel, samples sharing a letter are not significantly different (*P* > 0.05), *n*.*s*. indicates no significant differences among samples. (a) Average total N_2_O emissions for the measurement period (May-September) are given as back-transformed geometric means ± standard error. (b) Average sub-rooting zone NO_3_^-^ concentrations are given as back-transformed geometric means ± standard error. (c) Average aboveground productivity values are given as arithmetic means ± standard error.

Crop type and fertilizer effects on total N_2_O emissions were significant in both years (Fig 1A and 1B). Fertilization increased emissions in all cases except for native grasses in 2012, where it had no effect. With equal fertilization, N_2_O emissions were comparable between the polycultures and lower than switchgrass. In 2011, N_2_O emissions from all crop types were approximately 3 times greater from the fertilized plots when compared to the unfertilized plots (Fig 1A). Emission factors in 2011 were 2.4% (s.e. ± 0.65%), 0.26% (s.e. ± 0.13%), and 0.56% (s.e. ± 0.26%) for switchgrass, native grasses, and restored prairie, respectively. In 2012, nitrogen fertilization similarly tripled N_2_O emissions from switchgrass, but did not impact the polycultures (Fig 1B). Emission factors for 2012 were 1.5% (s.e. ± 0.48%), 0.018% (s.e. ± 0.041%), and 0.23% (s.e. ± 0.025%) for switchgrass, native grasses, and restored prairie, respectively.

### Potential nitrate leaching increased with fertilization in switchgrass

In both years, fertilized switchgrass had higher NO_3_^-^ concentrations below the rooting zone than the polycultures, while all other cropping system × fertilization combinations were comparable (Fig 1C and 1D). Across all systems, NO_3_^-^ concentrations were lower in 2012 than 2011. In 2011, there was no significant effect of fertilizer on potential leaching (Fig 1C). In 2012 there was a crop × fertilizer effect with higher potential leaching in fertilized versus unfertilized switchgrass, but no fertilizer effect in the polycultures (Fig 1D). Regardless of fertilizer use, early season soil NO_3_^-^ was higher in switchgrass than either polyculture, while later-season values converged among crops (S3 Fig). The exception was the unfertilized crops in 2012 where NO_3_^-^ values differed among all three systems in the early season but converged by peak biomass.

### Aboveground net primary productivity of polycultures increased by fertilization

Fertilization increased ANPP in polycultures, although the increase was only significant for restored prairie in 2011 and native grasses in 2012 (Fig 1E and 1F). In contrast, fertilization did not significantly increase switchgrass ANPP in 2011 and reduced it in 2012. Switchgrass was generally more productive than the polycultures, which were not significantly different from each other within the fertilization treatment.

**Fig 2:**
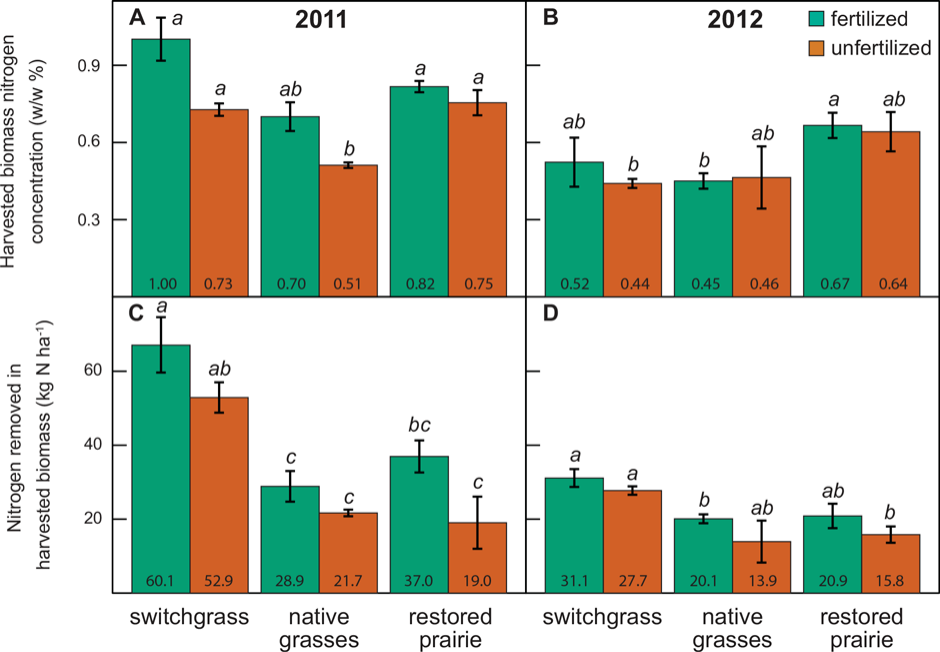
Harvested biomass properties for each crop and fertilizer treatment in 2011 and 2012. Within each panel, samples sharing a letter are not significantly different (*P* > 0.05). All values are arithmetic means ± standard error.

Fertilization did not increase the ratio of N_2_O emissions or sub-rooting zone NO_3_^-^ concentrations to ANPP (Table 2). In most cases, fertilized polycultures had significantly lower ratios than fertilized switchgrass. All systems had similar aboveground biomass N concentrations in 2011, while in 2012 restored prairie had the highest concentrations (Fig 2A and 2B). Fertilization increased aboveground N concentrations only in switchgrass and the native grasses, and then only in 2011. Fertilization increased N removed in harvested biomass for switchgrass in 2011 and restored prairie in both years (Fig 2C and 2D). Switchgrass had the greatest N removal in 2011, while in 2012 switchgrass and fertilized restored prairie had equivalent removal rates.

**Table 2:** Relationship between nitrogen loss and aboveground net primary productivity (ANPP) production in three bioenergy cropping systems.

			g N loss Mg^-1^ biomass					
Year	Crop	Fertilizer treatment	N_2_O			NO_3_^-^		
2011	Switchgrass	Fertilized	114	(94–139)	*a*	513	(295–892)	*a*
		Unfertilized	39	(31–48)	*ab*	312	(179–541)	*a*
	Native grasses	Fertilized	20	(17–25)	*b*	277	(162–472)	*a*
		Unfertilized	10	(7–13)	*b*	321	(185–559)	*a*
	Restored prairie	Fertilized	34	(26–43)	*ab*	253	(153–421)	*a*
		Unfertilized	12	(9–15)	*b*	391	(225–680)	*a*
2012	Switchgrass	Fertilized	125	(95–164)	*a*	483	(344–677)	*a*
		Unfertilized	29	(23–36)	*b*	71	(47–106)	*b*
	Native grasses	Fertilized	18	(15–22)	*b*	81	(51–129)	*ab*
		Unfertilized	26	(20–32)	*b*	130	(84–200)	*ab*
	Restored prairie	Fertilized	31	(26–38)	*b*	49	(33–73)	*b*
		Unfertilized	31	(16–27)	*b*	99	(61–161)	*ab*

Values are geometric means, with ± 1 s.e. in parentheses. Within a year and column, values sharing a letter are not significantly different (*P* > 0.05).

### Soil moisture and temperature

Average soil temperatures to 10 cm exhibited a crop × fertilizer interaction with significantly higher temperatures in 2012 than 2011 (S1 and S2 Tables). In native grasses and restored prairie systems, unfertilized subplots were warmer. Soil WFPS exhibited a crop × year effect and was consistently higher in unfertilized subplots (S1 and S2 Tables). Within each crop, WFPS in 2012 was lower than 2011. In 2011, WFPS in the native grasses was significantly lower than other crops while in 2012 WFPS in the native grasses was lower than restored prairie but not significantly different from switchgrass.

## Discussion

We observed a cropping systems-level tradeoff between biomass production and loss of environmentally harmful forms of nitrogen (N), with the native grasses and prairie species mixtures having lower N_2_O emissions, potential NO_3_^-^ leaching, and aboveground productivity. N losses resulting from fertilization were greater and more consistent in switchgrass than in the two polycultures, while productivity gains in response to fertilization were observed only in the polycultures. Both mechanisms of N loss we considered in this study are heavily influenced by precipitation and soil moisture; our measurements in the drought year of 2012 allowed us to compare the interplay of soil moisture and labile N availability across the three cropping systems. Given that all three systems were managed identically following best practices for switchgrass production, our findings suggest that perennial grass-based polycultures handle fertilizer-applied N more robustly and efficiently than a switchgrass monoculture.

We hypothesize that many of our findings result from differences in how quickly and completely the cropping systems we studied are able to immobilize applied N. This immobilization primarily entails incorporation of labile N into plant and microbial biomass [45]. Ideally, N fertilizer application should coincide with periods of heavy plant uptake, ensuring as much N as possible is immobilized into plant tissues [8]. Predicting phenology in perennial species is extremely challenging; in perennial bunch-forming grasses, like switchgrass, windows for conducting field operations are further constrained by the need to avoid damage to plant crowns. In this context, polycultures benefit greatly from seasonal complementarity, which has been demonstrated to reduce labile N levels in soil [25]. Polycultures incorporate species with a range of phenologies, so timing of plant N demand overlap to provide a broader time window for efficient N uptake. In addition, both polycultures in our study contained significant components of cool-season grasses and/or forbs. These taxa have higher N demands and greater early-season activity than a warm-season grass like switchgrass, and thus should have been well-positioned to assimilate applied N.

Our N application rates were low, approximately half the rate recommended by University of Wisconsin Extension for established switchgrass [8] and below the agronomically optimal fertilization rate for a mixture of prairie species grown for biomass [15]. Despite this, the amount of N we applied exceeded the amount removed as harvested biomass in all cases except for the fertilized switchgrass in 2011. One guideline for N management in bioenergy crops is to match application rates to the amount of N anticipated to be removed by biomass [46]. This calculation can be complicated, as many perennial species, including switchgrass, are capable of progressively relocating N from their aboveground biomass to their belowground tissues over the period of senescence [47]. The N content of biomass can thus potentially vary interannually, depending on when in the senescence process the plants were harvested, as we observed with broadly lower N contents in 2012. That said, we did not observe consistent, significant differences in biomass N concentrations across cropping systems. Rates of N removal were largely due to differences in harvested biomass. It is curious that the polycultures had a greater excess of N fertilization but lower levels of N loss. Most likely, much of the excess N was immobilized into microbial biomass. Prairies in south-central Wisconsin support greater microbial biomass than switchgrass fields [48], and in these soils biomass and necromass constitute a significant component of the organic N pool [49]. It would be important to investigate the practical limits of organic N accumulation in soils, as these would determine how these polycultures would behave with long-term excessive N application.

Concentrations of NO_3_^-^ at 50–80 cm responded strongly to cropping systems, N application, and yearly precipitation dynamics. These concentrations were dramatically higher in the switchgrass system early in the season, decreasing sharply over time. Notably, while fertilization increased concentrations, the dynamic was still present in unfertilized switchgrass, indicating considerable free NO_3_^-^ in the system early in the season. Given the limited root activity occurring at these depths [34,35], these decreases likely represent loss of NO_3_^-^ from the system, rather than growing season uptake by plants. Concentrations were much lower in 2012, where the lack of precipitation limited the percolation of water needed to transport NO_3_^-^. The contrast to the polycultures is stark and informative. In both systems, NO_3_^-^ concentrations did not increase in the early season, did not increase with fertilization, and were largely the same in the normal and drought years. These observations strongly suggest that, even following fertilization in a year with normal precipitation, soil NO_3_^-^ levels in the polycultural systems were low enough to avoid significant leaching. This demonstrated the capacity of these polycultural systems to rapidly and efficiently immobilize fertilizer N.

Patterns of N_2_O emissions similarly reflected differences in N immobilization efficiency across cropping systems, fertilization managements, and years. Soils can produce N_2_O through both the processes of nitrification and denitrification, but in soils with adequate moisture, such as at our study site, flux dynamics are dominated by denitrification [50]. Denitrification uses free NO_3_^-^ as an alternative electron acceptor under low oxygen conditions. Typically, large N_2_O fluxes are observed when precipitation events, which reduce oxygen availability by restricting gas movement in the soil, coincide with high concentrations of NO_3_^-^, which is usually the limiting substrate for the reaction [27]. The lower N_2_O emissions observed in the fertilized polycultures in the normal precipitation year of 2011 illustrate how effectively these systems were able to immobilize N between the time it was applied and the next major precipitation event. The lack of spring precipitation in 2012, by contrast, provided greater time for N immobilization. This was sufficient to completely erase the fertilizer effect in the polycultures, but the effect was still clearly visible in the switchgrass, suggesting that the systems differed both in the speed and the thoroughness of their N immobilization. It should be noted that in 2011 switchgrass had slightly higher soil moisture, potentially accounting for some of the increase in emissions, but soil moistures were comparable across systems in 2012 while emission differences remained.

In light of the N immobilization dynamics we observed, the productivity responses to N fertilization suggest the polycultures were mildly N-limited, while the switchgrass was not. Switchgrass responds unevenly to N fertilization [51], which can even decrease switchgrass productivity under high soil N conditions [52]. Fertilization may indirectly inhibit switchgrass growth by stimulating forb and cool-season grass weeds, which can be detrimental to switchgrass productivity [53] and stand establishment [13,14]. The polycultures, by contrast, deliberately included highly productive forbs and cool-season grasses, whose stimulation by N addition contributed to the system's productivity. It needs to be noted, however, that fertilized and unfertilized switchgrass were at least as productive as the polycultures, as has been previously reported [54]. The polycultures juxtaposed low N losses with productivity gains in response to fertilization, suggesting these systems were N limited. It is possible that the microbial biomass in these systems competed with the plants for nitrogen [55], an interaction that may complicate management of these systems.

Our findings hold significant implications for the selection and management of perennial bioenergy cropping systems. Selecting a bioenergy cropping system may entail balancing biomass production needs with N pollution risks, although productivity gains through optimization of polyculture species assemblages may be possible. Our results show, with respect to nitrogen processing, polycultural biomass production systems can be resilient in the face of annual weather fluctuations which indicates the environmental and management benefits of these systems should not be overlooked.

## Supporting Information

S1 Fig High-frequency soil and climate measurements collected from a single switchgrass plot in the Bioenergy Cropping Systems Experiment at Arlington Agricultural Research Station. (PDF)

S2 Fig Biofuels Cropping System Experiment (BCSE) plot dimensions and sampling design. (PDF)

S3 Fig Growing season soil nitrate concentrations at 50 to 80 cm. (PDF)

S1 Table Mean growing season soil temperature and water filled pore space (WFPS) to 10 cm by cropping system, year, and fertilization. (PDF)

S2 Table Variance partitioning of effects of year, cropping system, and fertilization on mean growing season soil temperature and water filled pore space (WFPS) to 10 cm. (PDF)
