# Supplementary material for: Nitrogen Fertilization Effects on Productivity and Nitrogen Loss in Three Grass-Based Perennial Bioenergy Cropping Systems
Source: PLoS One. 2016 Mar 18;11(3):e0151919. doi: 10.1371/journal.pone.0151919 (PMC4798553; doi:10.1371/journal.pone.0151919)
Supplement: S1 Fig — (PDF) [file pone.0151919.s001.pdf]

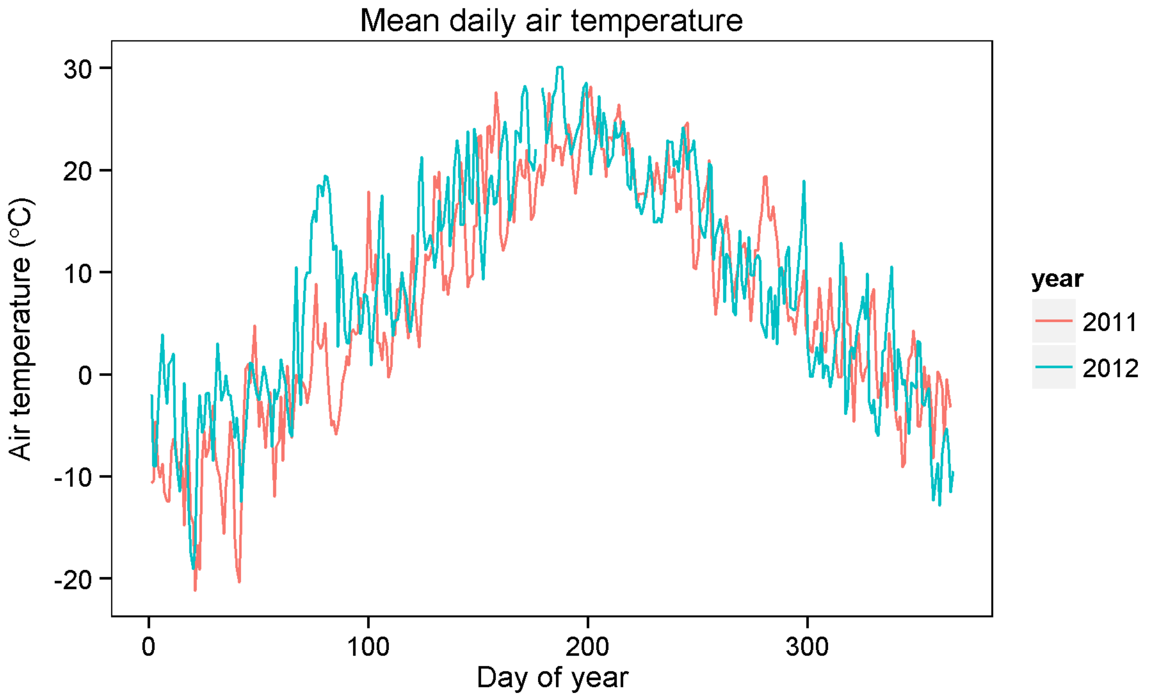
Mean daily air temperature
Air temperature (°C)
30
20
10
0
-10
-20
0
100
200
300
Day of year
year
2011
2012

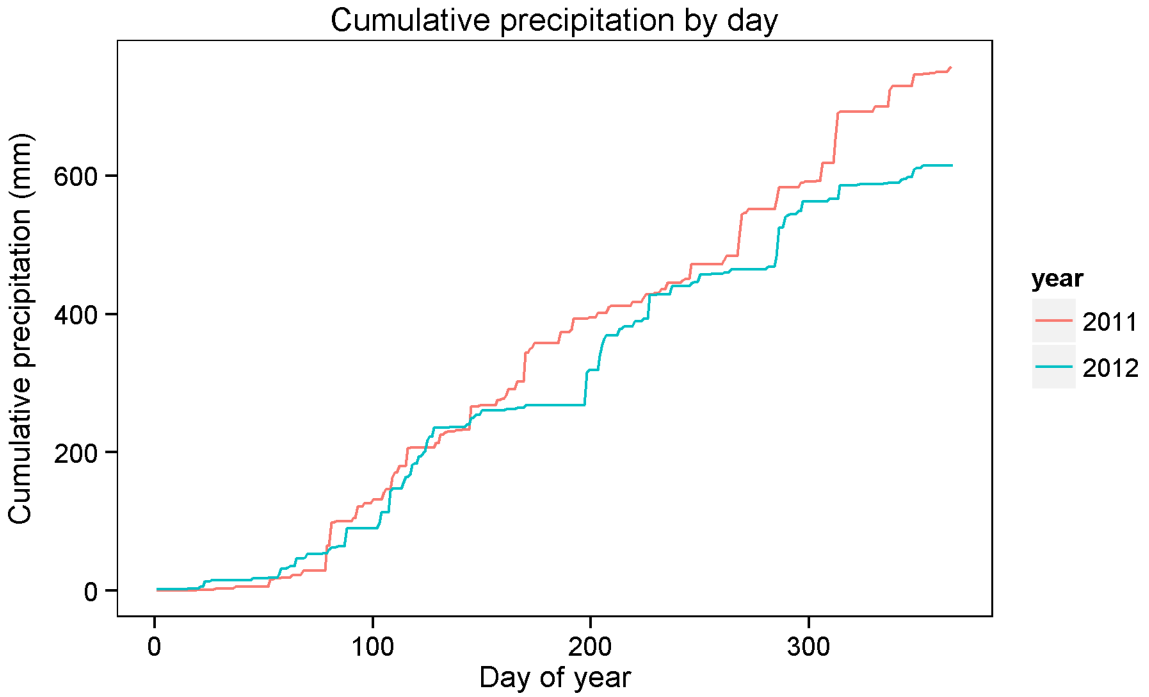
Cumulative precipitation by day
Cumulative precipitation (mm)
600
400
200
0
0
100
200
300
Day of year
year
2011
2012

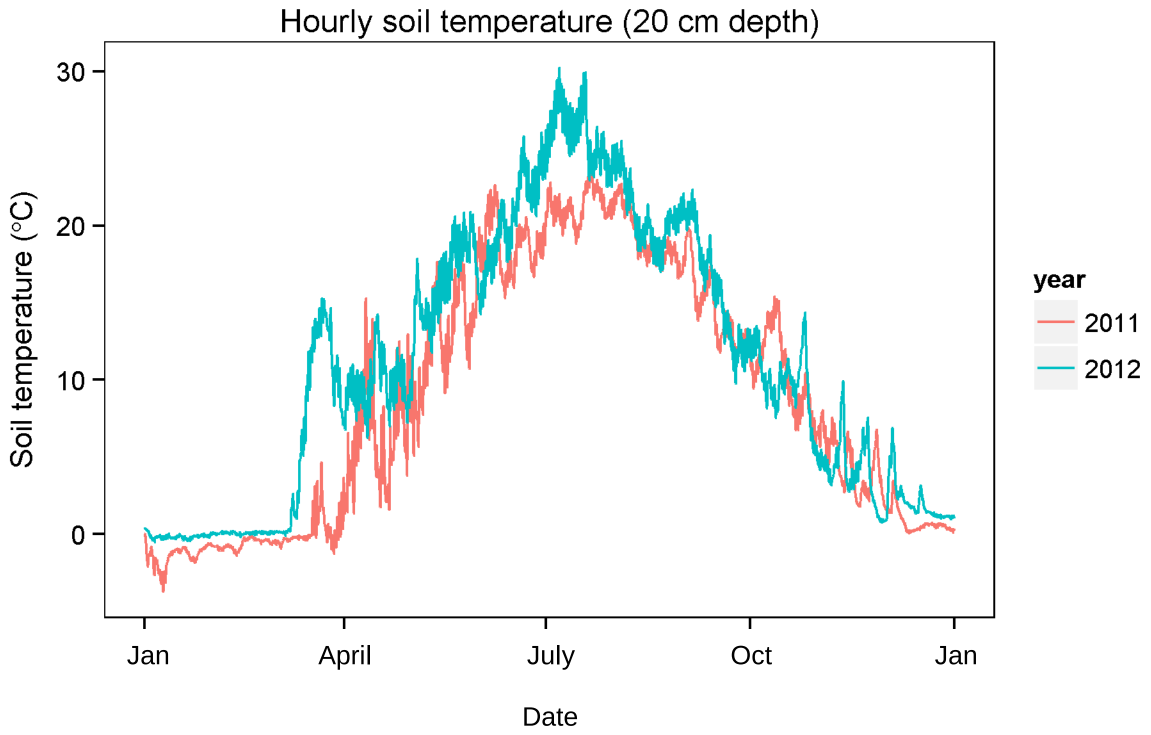
Hourly soil temperature (20 cm depth)
Soil temperature (°C)
30
20
10
0
Jan
April
July
Oct
Jan
Date
year
2011
2012

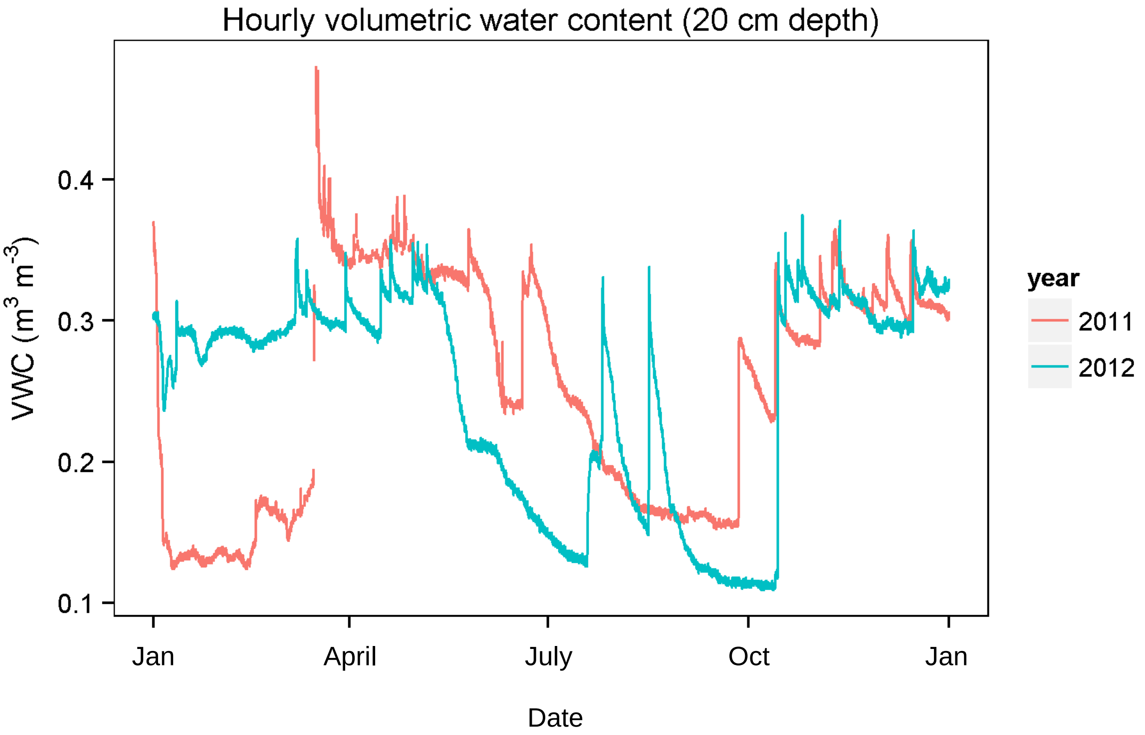
Hourly volumetric water content (20 cm depth)
VWC (m3 m-3)
0.4
0.3
0.2
0.1
Jan
April
July
Oct
Jan
Date
year
2011
2012

S1 Figure. High-frequency soil and climate measurements collected from a single switchgrass plot in the Bioenergy Cropping Systems Experiment at Arlington Agricultural Research Station.
