# Supplementary figures and images for: Nitrogen Fertilization Effects on Productivity and Nitrogen Loss in Three Grass-Based Perennial Bioenergy Cropping Systems
Source: PLoS One. 2016 Mar 18;11(3):e0151919. doi: 10.1371/journal.pone.0151919 (PMC4798553; doi:10.1371/journal.pone.0151919)

## BCSE plot dimensions and sample design

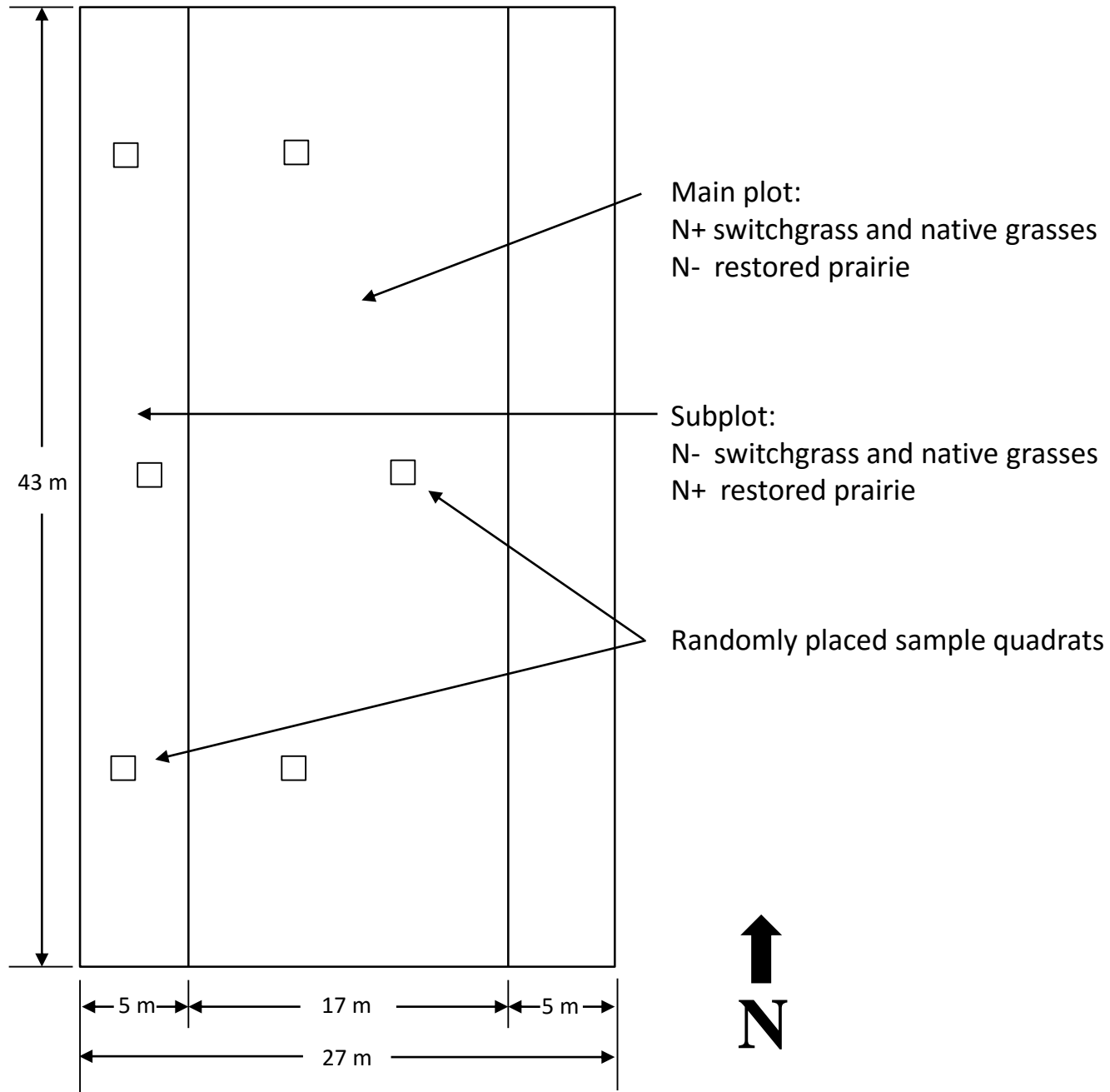

Supplement: S2 Fig — (PDF) [file pone.0151919.s002.pdf]
