# Supplementary material for: Nitrogen Fertilization Effects on Productivity and Nitrogen Loss in Three Grass-Based Perennial Bioenergy Cropping Systems
Source: PLoS One. 2016 Mar 18;11(3):e0151919. doi: 10.1371/journal.pone.0151919 (PMC4798553; doi:10.1371/journal.pone.0151919)
Supplement: S3 Fig — (PDF) [file pone.0151919.s003.pdf]

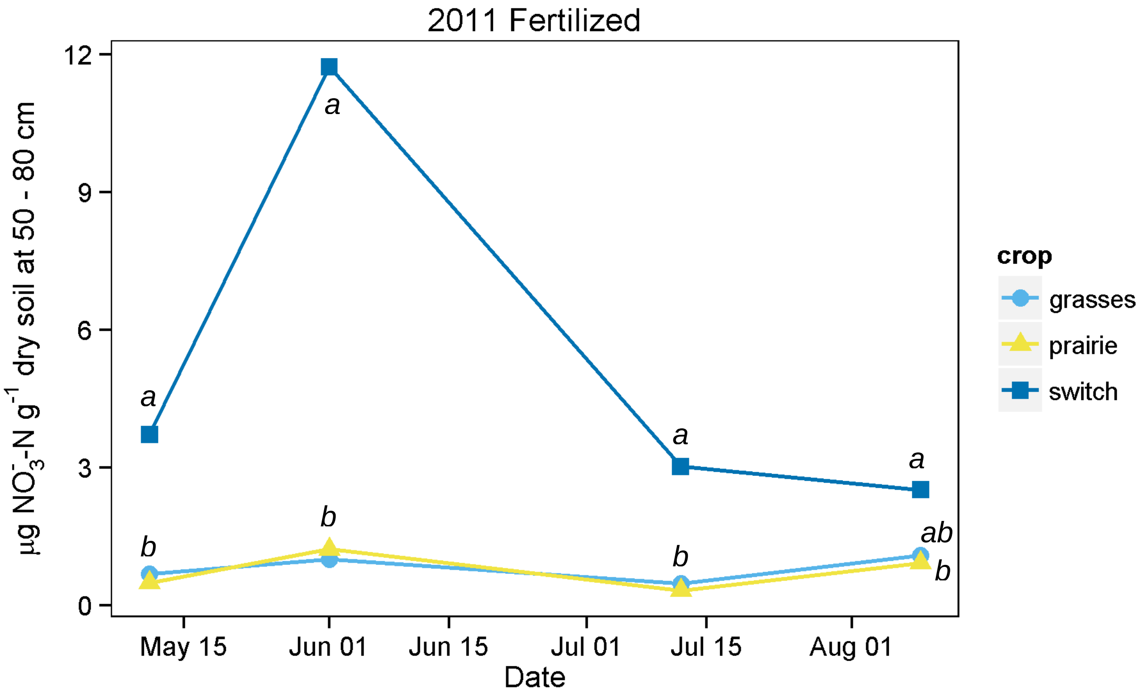
2011 Fertilized
μg NO3--N g-1 dry soil at 50 - 80 cm
Date
May 15
Jun 01
Jun 15
Jul 01
Jul 15
Aug 01
crop
grasses
prairie
switch
a
b
ab

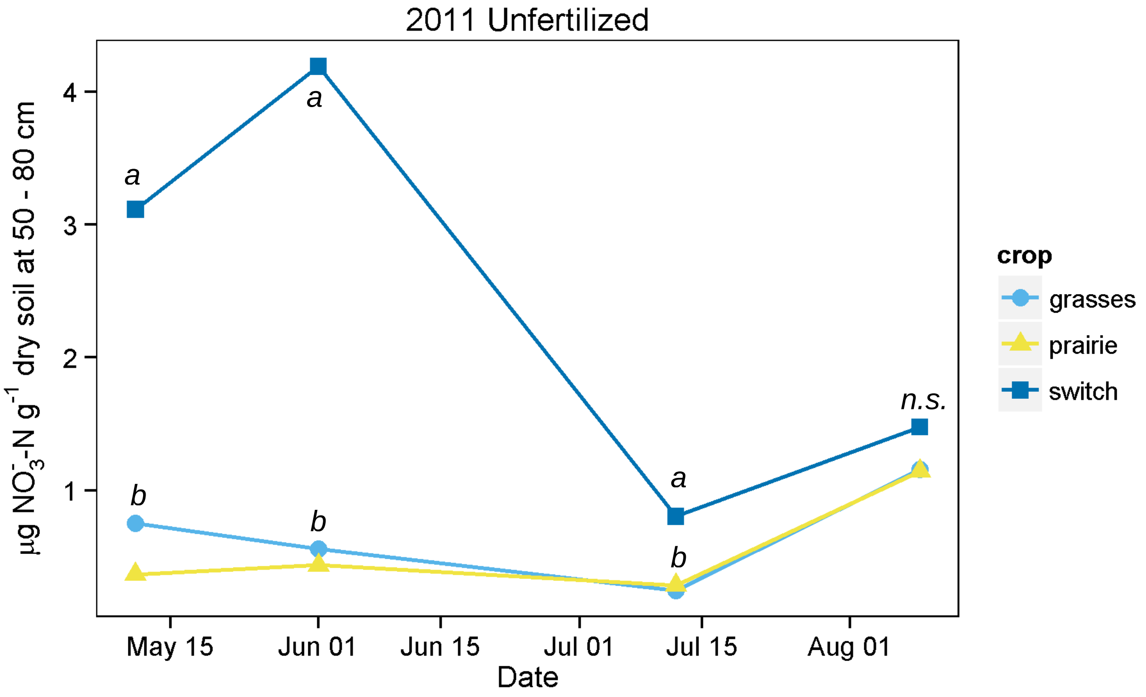
2011 Unfertilized
μg NO3--N g-1 dry soil at 50 - 80 cm
Date
May 15
Jun 01
Jun 15
Jul 01
Jul 15
Aug 01
crop
grasses
prairie
switch
a
b
n.s.

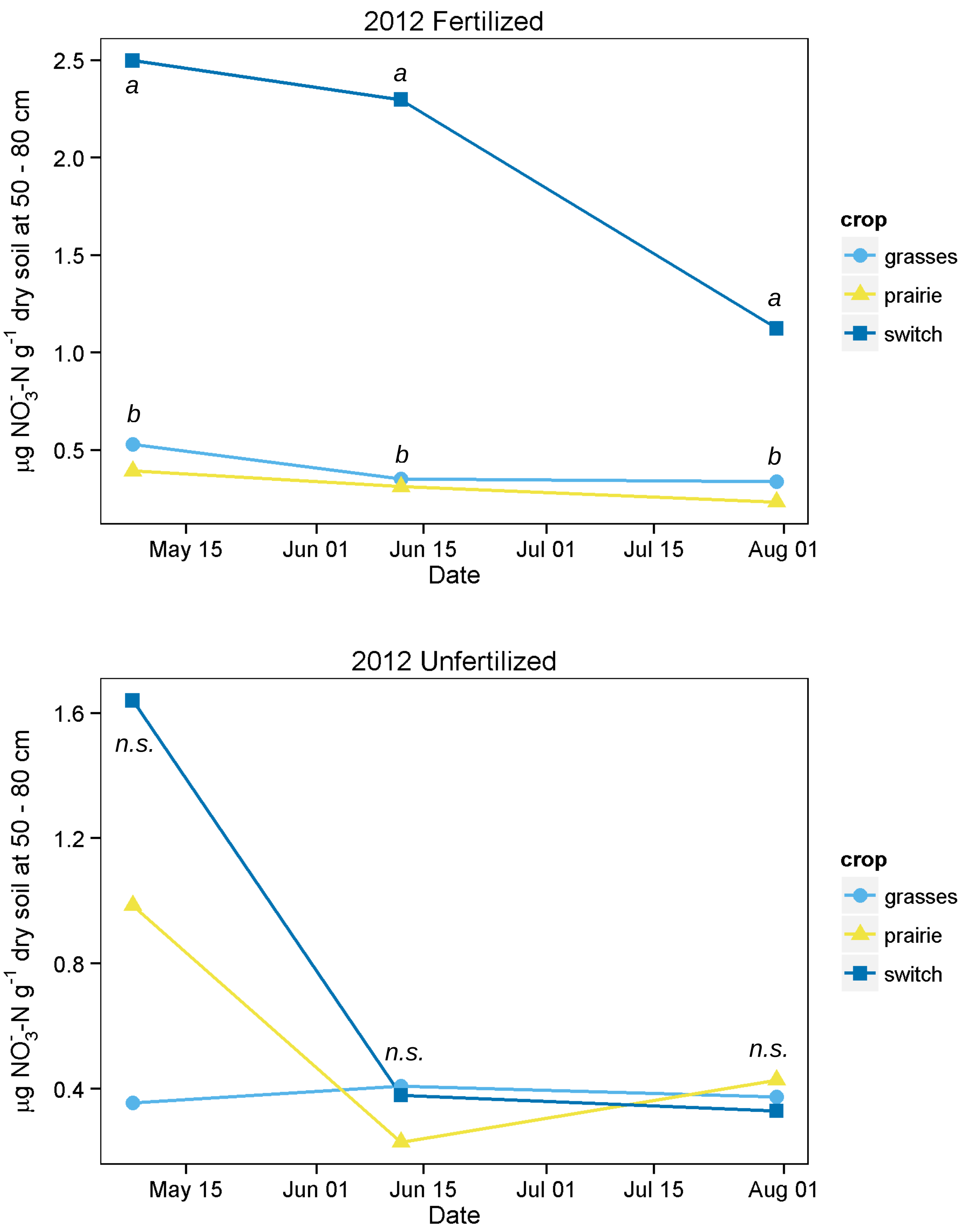

S2 Figure. Growing season soil nitrate concentrations. Plotted values are treatment means. Letters indicate groups not significantly different (*P* > 0.05 after Tukey correction), n.s. indicates no significant differences among crops on a date.
