# Supplementary material for: Nitrogen Fertilization Effects on Productivity and Nitrogen Loss in Three Grass-Based Perennial Bioenergy Cropping Systems
Source: PLoS One. 2016 Mar 18;11(3):e0151919. doi: 10.1371/journal.pone.0151919 (PMC4798553; doi:10.1371/journal.pone.0151919)
Supplement: S1 Table — (PDF) [file pone.0151919.s004.pdf]

S1 Table. Mean growing season soil temperature and water filled pore space (WFPS) to 10 cm by cropping system, year, and fertilization.

Cropping system	Year	Fertilization	Soil temp. (ºC)	Water-filled pore space (%)
Switchgrass	2011	Fertilized	19.3 ± 0.2	40.5 ± 1.3
		Unfertilized	19.4 ± 0.2	41.1 ± 1.3
	2012	Fertilized	20.9 ± 0.2	34.8 ± 1.0
		Unfertilized	20.8 ± 0.2	36.2 ± 1.0
Native grasses	2011	Fertilized	19.3 ± 0.2	35.5 ± 1.3
		Unfertilized	19.7 ± 0.2	36.3 ± 1.3
	2012	Fertilized	20.8 ± 0.2	32.5 ± 1.0
		Unfertilized	21.2 ± 0.2	35.0 ± 1.0
Restored prairie	2011	Fertilized	19.3 ± 0.2	38.2 ± 1.3
		Unfertilized	20.1 ± 0.2	39.8 ± 1.3
	2012	Fertilized	20.8 ± 0.2	34.9 ± 1.0
		Unfertilized	21.3 ± 0.2	36.9 ± 1.0

Values are means (± s.e.) over all measurements taken during the May-September growing season.
