# Supplementary material for: Nitrogen Fertilization Effects on Productivity and Nitrogen Loss in Three Grass-Based Perennial Bioenergy Cropping Systems
Source: PLoS One. 2016 Mar 18;11(3):e0151919. doi: 10.1371/journal.pone.0151919 (PMC4798553; doi:10.1371/journal.pone.0151919)
Supplement: S2 Table — (PDF) [file pone.0151919.s005.pdf]

S2 Table. Variance partitioning of effects of year, cropping system, and fertilization on mean growing season soil temperature and water filled pore space (WFPS) to 10 cm.

	Water-filled pore space				Soil temperature			
	F	$d.f._{num}$	$d.f._{den}$	*P*	*F*	$d.f._{num}$	$d.f._{den}$	*P*
Fertilization (F)	19.1	1	6	0.005 **	5.4	1	6	0.059
Crop (C)	3.3	2	4	0.142	1.6	2	4	0.317
Year (Y)	61.8	1	84	<0.001 ***	197.2	1	84	<0.001 ***
F × C	0.5	2	6	0.633	2.0	2	6	0.216
F × Y	1.0	1	84	0.312	0.9	1	84	0.351
C × Y	4.2	2	84	0.018 *	0.1	2	84	0.869
F × Y × C	0.2	2	84	0.811	0.3	2	84	0.744

ANOVA based on a linear mixed effects model with a random effect of fertility treatment within plot within block. $d.f._{num}$ is numerator degrees of freedom, $d.f._{den}$ is denominator degrees of freedom.
* $P < 0.05$, ** $P < 0.01$, *** $P < 0.001$
